# Use of off-label compounded oral sildenafil in the management of persistent pulmonary hypertension of the newborn: A case report

**DOI:** 10.34172/jcvtr.2022.03

**Published:** 2022-03-07

**Authors:** Olapeju Daniyan, Obumneme Ezeanosike, Dorathy Obu, Fortune Ujunwa

**Affiliations:** ^1^Department of Paediatrics, Alex Ekwueme Federal University Teaching Hospital, Abakaliki, Ebonyi State, Nigeria; ^2^Department of Paediatrics, Ebonyi State University, Abakaliki, Ebonyi State, Nigeria; ^3^Department of Paediatrics, College of Medicine University of Nigeria, Enugu Campus, Enugu, Nigeria

**Keywords:** Newborn, Persistent Pulmonary Hypertension, Sildenafil

## Abstract

We report a case of a newborn with persistent pulmonary hypertension (PPHN) due to meconium aspiration syndrome with associated lung collapse. Echocardiogram revealed features of persistent pulmonary hypertension. He was treated with compounded oral sildenafil. Oral sildenafil has proven to be effective and safe in the management of PPHN in neonates with persistent pulmonary hypertension. Therefore, in situations where inhaled nitric oxide is not available it may be used as an alternative therapy in PPHN. Further randomized controlled studies are needed to determine its efficacy and pharmacokinetics.

## Introduction

 Persistent pulmonary hypertension of the newborn (PPHN) occurs when there is failure of reduction of the pulmonary vascular pressure after birth,^1^ this results in right- to-left shunting of blood through the foramen ovale or ductus arteriosus. During intrauterine life there is high pulmonary vascular pressure which results in decreased blood flow through the pulmonary artery (< 20 %), a large percentage of blood being diverted from the lungs through the ductus arteriosus.^2^ Multiple factors play roles in maintaining a high pulmonary artery pressure in-utero and these include low oxygen tension, fetal lung fluid, production of endothelin-1 which is a vasoconstrictor mediator and inhibition of nitric oxide and prostaglandin.^3^ At birth when the first breath is taken, the lungs are filled with air, fluid in the lungs is gradually replaced by the air containing oxygen which leads to pulmonary vasodilatation. This results in a decrease in pulmonary artery pressure and an increase in the blood flow through the pulmonary artery to the lungs, this process completes by two weeks post-natally. Other factors that contribute to the decrease of pulmonary vascular pressure include the production of prostaglandin E_1 _and nitric oxide secretion.^4^

 Prevalence of persistent pulmonary hypertension of the newborn is estimated to be 1.9 per 1000 births in the United States^5^ while a prevalence rate of 1.1% was reported in South Africa.^6^ Meconium aspiration syndrome is one of the major causes of PPHN,^7^it leads to obstruction of the airway which results in atelectasis and lung collapse.^8^ Treatment of PPHN includes the use of vasodilators like inhaled nitric oxide, prostaglandins and sildenafil. Inhaled nitric oxide is not readily available in resource limited countries such as ours, whereas sildenafil is available and has been reported to be effective and safe when given either by intravenous or oral route.^7^ There is dearth of literature from Nigeria on PPHN and the use of compounded oral sildenafil. This case report is important to create awareness among physicians managing newborns especially in developing countries who may encounter challenges managing newborns with persistent pulmonary hypertension due to non-availability of drugs like inhaled nitric oxide. Sildenafil which is readily available can be used in place of inhaled nitric oxide to improve outcome in these newborns. The authors report a case of persistent pulmonary hypertension of the newborn that was successfully treated with oral sildenafil.

## Case Presentation

 A 2-day old male neonate was admitted into the newborn special care baby unit following a referral from a peripheral hospital on account of poor cry at birth and difficulty in breathing noticed few minutes after birth. The baby was delivered to a 28-year old primiparous mother at 41 weeks gestation through emergency caesarean section due to prolonged obstructed labour, the liquor was stained with meconium. The APGAR score at birth was given as 5 at 1st min and 6 at 5th min. Mother had antenatal care at a primary healthcare facility, she had no history of febrile illness or antepartum bleeding in pregnancy. She was not a known hypertensive or diabetic. She did not take any other medications in pregnancy except the routine antenatal care medications like folic acid and fersolate.

 Examination findings on presentation included severe respiratory distress, central cyanosis, lethargy, hypertonia. He had a respiratory rate of 90 cycles/min and a heart rate of 138 beats/min. His weight was 3.1kg, length was 35cm, head circumference 36cm. His primitive reflexes were suboptimal and the umbilical stump was stained with meconium. Respiratory examination revealed bulging of the anterior chest wall and bilateral coarse crepitation in the lung fields. Oxygen saturation (SpO_2)_ ranges between 55 to 72%.

**Figure 1:**
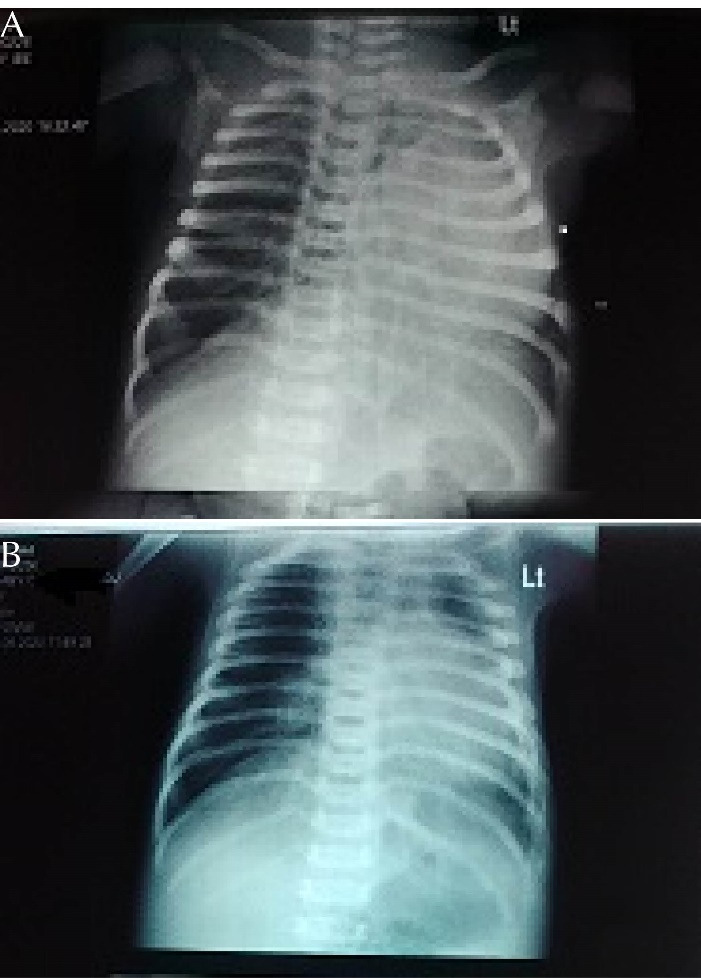
(A): Chest radiograph showing left lung collapse, (B): improvement in lung markings after commencement of oral Sildenafil

 He was commenced on intranasal oxygen and subsequently placed on continuous positive airway pressure, intravenous fluid and intravenous antibiotics. The chest radiograph showed left lung collapse from possible left main bronchus obstruction and compensatory hyperinflation on the right lung (Figure 1A). A 2D Echocardiography showed moderate tricuspid regurgitation, pulmonary artery pressure of 30mmHg with a right-to-left shunt through a patent foramen ovale and left atrial dialatation

 The patient was commenced on compounded oral sildenafil 1mg/kg per dose every 6 hours given through nasogastric tube, oral enalapril at 0.08mg/kg daily and iv frusemide 0.5mg/kg per dose every 12 hours. The oral sildenafil was compounded in the hospital pharmacy.

 There was a significant improvement in the respiratory distress by the 3rd day following commencement of these medications. The SpO2 improved from 72% to 96% while the respiratory rate reduced from 90 cycles/min to 62 cycles/min. A repeat 2D ECHO done two weeks after initiation of oral sildenafil showed a reduction in the pulmonary artery pressure to 20mmHg. While repeat chest radiograph showed improvement in the lung markings (Figure 1B). The baby was subsequently weaned off CPAP and continued on intranasal oxygen for few days before oxygen was discontinued. The baby was discharged home with follow up at both the Neonatology and Cardiology clinics.

## Discussion

 Persistent pulmonary hypertension of the newborn occurs when pulmonary vascular pressure remains elevated at birth. It is associated with severe respiratory distress and hypoxemia^9^ which was observed in the index patient. Risk factors for PPHN include male gender, African or Asian maternal race, maternal diabetes, maternal asthma,late preterm and large for gestational age and chorioamnionitis.^10^ The causes of PPHN has been classified into idiopathic, lung parenchyma disease (e.g meconum aspiration syndrome), abnormal transition at birth, developmental lung disease(e.g hypoplastic pulmonary vasculature ).^11^Meconium aspiration syndrome leads to mechanical obstruction which may result in atelectasis or lung collapse, chemical pneumonitis, inactivation of surfactant and pulmonary vasoconstriction.^12^ Diagnosis of PPHN is by echocardiography findings of right ventricular hypertrophy, deviation of the interventricular septum to the left, tricuspid regurgitation and right to left or bidirectional shunting at the patent foramen ovale and patent ductus arteriosus.^13^

 Inhaled nitric oxide, a pulmonary vasodilator is the main stay of treatment in PPHN^14^, but it is not readily available in developing countries because it is expensive and requires expertise to administer. Other vasodilators that have been used in PPHN include phosphodiesterase inhibitors e.g sildenafil ^15^ endothelin receptor antagonist^16^and synthetic prostacyclin analogues e.g iloprost.^17^ Sildenafil is a phosphodiesterse 5 inhibitor, it causes an increase in cyclic guanosine monophosphate ( cGMP) and can be administered orally or intravenously and has been shown to improve outcomes of neonates in resource- limited settings where inhaled nitric oxide is not available.^18^ Oral sildenafil was used in this patient because it was readily available and affordable. It is currently off-label^19^ in the newborn both for age, dose and for this indication in Nigeria. Off-label prescribing is when drugs are prescribed and used outside their licensed indications with respect to dosage, age, indication, or route.^20^ Oral Sildenafil currently comes as 50mg solid tablets, a formulation and dose that is inappropriate for the newborn, hence, to use the drug, it had to be compounded by the hospital pharmacist.

 There were no complications observed during its use in the index patient and serum electrolytes were within normal ranges. Vargas-Origel et al^15^reported improvement in oxygen parameters after 7 hours of sildenafil treatment compared to the placebo group with higher mortality among the placebo group. While in the index patient improvement in oxygen saturation was observed by the 3rd day of initiating oral sildenafil. These findings have shown that in developing countries where inhaled nitric oxide is not readily available sildenafil can be used in the treatment of PPHN with a good outcome. Early diagnosis using a 2D echocardiography is essential for early institution of therapy and good outcome in patients with PPHN.

## Conclusion

 Oral sildenafil has proven to be effective and safe in the management of PPHN in neonates with persistent pulmonary hypertension. Further randomized controlled studies are needed to determine its efficacy and pharmacokinetics.

## Acknowledgements

 Special thanks to Pharmacist Oko Christopher, Pharmacy Department of Alex Ekwueme Federal University Teaching Hospital Abakaliki, for compounding the oral sildenafil used in this patient.

## Funding

 None.

## Ethical approval

 Ethical approval was obtained from the ethical committee of Alex Ekwueme Federal University Teaching Hospital Abakaliki, with ethical number: AEFUTHA/REC/VOL3/2021/207. Informed consent was also obtained from the parents of the patient.

## Competing interests

 None declared.
